# Trends in the Recent Patent Literature on Cholinesterase Reactivators (2016–2019)

**DOI:** 10.3390/biom10030436

**Published:** 2020-03-12

**Authors:** Alexandre A. de Castro, Letícia C. Assis, Flávia V. Soares, Kamil Kuca, Daniel A. Polisel, Elaine F. F. da Cunha, Teodorico C. Ramalho

**Affiliations:** 1 Department of Chemistry, Federal University of Lavras, Lavras 37200-000, Brazil; alexandre.a.castro@hotmail.com (A.A.d.C.); leticiaassisquimica@hotmail.com (L.C.A.); flaviavillela09@yahoo.com.br (F.V.S.); dpolisel@yahoo.com.br (D.A.P.); elaine_cunha@ufla.br (E.F.F.d.C.); 2 Department of Chemistry, Faculty of Science, University of Hradec Kralove, 500 03 Hradec Kralove, Czech Republic

**Keywords:** organophosphorus compounds, acetylcholinesterase, therapeutic potential, reactivation process, new trends in reactivators

## Abstract

Acetylcholinesterase (AChE) is the key enzyme responsible for deactivating the ACh neurotransmitter. Irreversible or prolonged inhibition of AChE, therefore, elevates synaptic ACh leading to serious central and peripheral adverse effects which fall under the cholinergic syndrome spectra. To combat the toxic effects of some AChEI, such as organophosphorus (OP) nerve agents, many compounds with reactivator effects have been developed. Within the most outstanding reactivators, the substances denominated oximes stand out, showing good performance for reactivating AChE and restoring the normal synaptic acetylcholine (ACh) levels. This review was developed with the purpose of covering the new advances in AChE reactivation. Over the past years, researchers worldwide have made efforts to identify and develop novel active molecules. These researches have been moving farther into the search for novel agents that possess better effectiveness of reactivation and broad-spectrum reactivation against diverse OP agents. In addition, the discovery of ways to restore AChE in the aged form is also of great importance. This review will allow us to evaluate the major advances made in the discovery of new acetylcholinesterase reactivators by reviewing all patents published between 2016 and 2019. This is an important step in continuing this remarkable research so that new studies can begin.

## 1. Introduction

The organophosphorus (OP) compounds are part of a very important organic class of phosphorus-based molecules. A range of these toxic substances possesses significant domestic and industrial applications [1]. The OP agents were intensively employed as warfare nerve agents in the past wars, for instance, World War II. In this regard, the most dangerous and toxic to humans are well-known nerve agents. Furthermore, these substances have remarkable importance for agricultural purposes. For instance, pesticides play an important role in combating the pests that cause damages to agricultural crops so that it is possible to enhance productivity [2]. Despite the important application of these compounds, their toxic effects are extremely harmful to humans, animals, and the environment, with high contamination rates. 

Poisoning by OP may take place through skin contact, oral, and the respiratory tract. These molecules act by inhibiting the acetylcholinesterase (AChE) enzyme. This inhibition process is irreversible in the case of no immediate treatment, resulting in a prolonged inhibition. AChE is responsible for the hydrolysis of the acetylcholine (ACh) neurotransmitter, thus balancing the synaptic activity [3]. Due to AChE inhibition, the neurotransmitter accumulates into central and peripheral cholinergic sites, leading to the over-stimulation of cholinergic receptors. The major intoxication-related symptoms are excessive salivation, lacrimation, sweating, broncho-constriction, and neuromuscular block. The latter especially affects the muscles responsible for breathing, and consequently leads to death [3,4,5]. 

There are some indications that about 3 million cases of OP poisoning occur in the world each year [6,7]. Occasionally, there is the occurrence of terrorist attacks, such as that in Syria, in 2013, where Sarin terribly affected the civilians [8]. The huge number of OP poisoning cases and the big stocks of still available nerve agents in diverse countries make necessary the discovery of large OP broad-spectrum antidotes [9,10,11,12]. Currently, the treatment procedure for OP poisoning consists of the use of two classes of drugs: competitive muscarinic receptor antagonist, such as atropine, and the use of a reactivating substance, usually one agent from the oxime class [13,14,15]. Unfortunately, there is no universal antidote to date, and a broad-spectrum oxime capable of reactivating all types of OP-inhibited AChE/butyrylcholinesterase (BChE) is highly desired [15,16,17]. In this review, the recent advances in the development of novel antidotes and therapies were analyzed according to the patents produced in the past few years. 

## 2. Cholinesterase Enzymes–AChE and BChE

**Figure 1:**
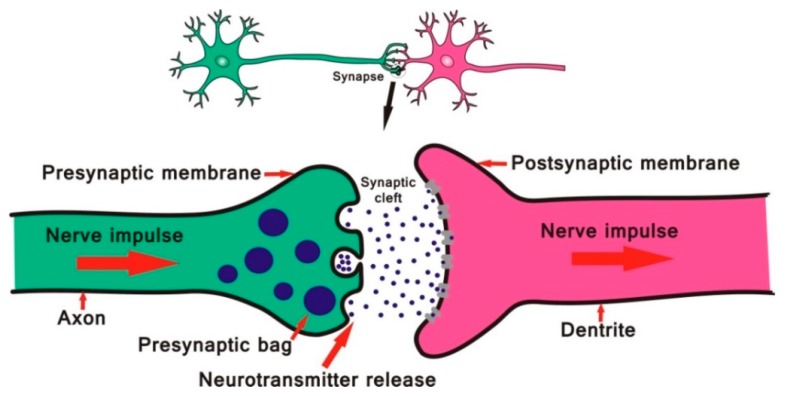
Neuron structure and nerve impulse transmission process.

Found in various parts of the body, such as neuromuscular junctions in the peripheral nervous system (PNS), parasympathetic nervous system (PSNS), central nervous system (CNS) synapses, and linked to erythrocyte membranes in the blood, AChE plays a fundamental role in the neurosynaptic communication process (Figure 1) [18].

**Figure 2:**
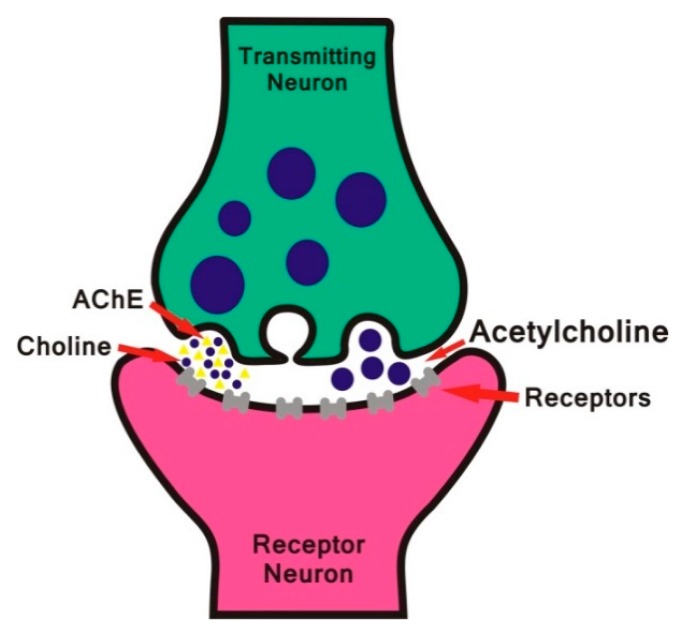
Representation of the acetylcholine (ACh) hydrolysis scheme.

With a determining action in the finalization of the nerve impulse propagation, AChE is responsible for maintaining the appropriate levels of ACh (Figure 2) [19]. AChE inactivates the action of ACh by hydrolyzing it into choline and acetate [20].

The interaction of AChE with the neurotransmitter takes place through two essential spots. The first one is the anionic site, where there is an interaction between the nitrogen positive charge of ACh and the negative charge produced by the aspartate residue. The second one is the stearic site, where the ACh ester group performs hydrogen bonding with the tyrosine residue [21,22,23]. The anionic region serves to guide the substrate to the position necessary to undergo the hydrolysis process [24]. The peripheral anionic site (PAS), located at a distance equal to or greater than 4.7 Å from the hydroxyl group of Ser203, guides and accommodates the quaternary nitrogen of the ACh molecule that enters the active site, electrostatically interacting with Glu334 [25]. 

In addition, the butyrylcholinesterase (BChE) enzyme, such as AChE, is another cholinesterase enzyme capable of hydrolyzing choline-based esters [26]. At the molecular level, the main difference between these cholinesterases is the fact that BChE lacks six aromatic amino acids out of the fourteen that line the AChE catalytic gorge [27,28]. Thus, the BChE gorge almost doubles the width, making the BChE active site domain more accessible to a wide variety of substrates and inhibitors [29,30]. While AChE acts preferentially by hydrolyzing ACh, BChE hydrolyzes both ACh and butyrylcholine (BCh) in similar amounts [31]. However, AChE is the enzyme with the highest catalytic efficiency known today, with an enzymatic hydrolysis rate of approximately 5.000 ACh molecules/s^4^ [32]. 

## 3. AChE Inhibition Processes

There are several drugs that target cholinergic synapses by inhibiting or reactivating AChE [26]. Anticholinesterase is the term used to name AChE inhibitor (AChEI) drugs. The mechanism of action of these inhibitors involves the competitive blockade of the AChE enzyme, prolonging the duration and intensity of ACh at the synaptic terminals. Regarding their binding to AChE, anticholinesterases can be classified as reversible, irreversible, and pseudo-irreversible. 

**Figure 3:**
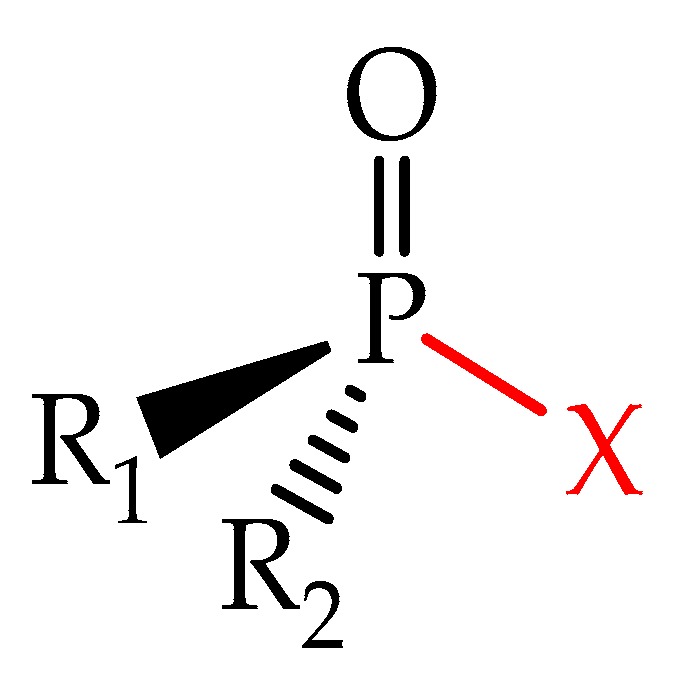
General structure of organophosphorus compounds.

Irreversible anticholinesterase agents are pentavalent phosphorus compounds that contain a labile group, such as fluoride, or an organic group [33]. Their general chemical structure can be observed in Figure 3. These agents spontaneously phosphorylate AChE, making OP poisoning very dangerous. The cholinesterase-inhibiting pesticides are well absorbed by all digestive, respiratory, and dermal routes. This property is due to their high fat solubility. They are biotransformed by oxidases, hydrolases, and transferases enzymes, whose process occurs mainly by hydrolysis, oxidation, and conjugation with glutathione. After absorption, they are rapidly and widely distributed to various tissues and organs, reaching higher concentrations in the liver and kidneys. Some highly lipophilic OPs deposit in adipose tissues and are gradually released over several days after exposure. Some works have been performed to employ the enzymatic biodegradation of these compounds by using different degrading enzymes [34,35,36,37,38,39,40].

**Figure 4:**
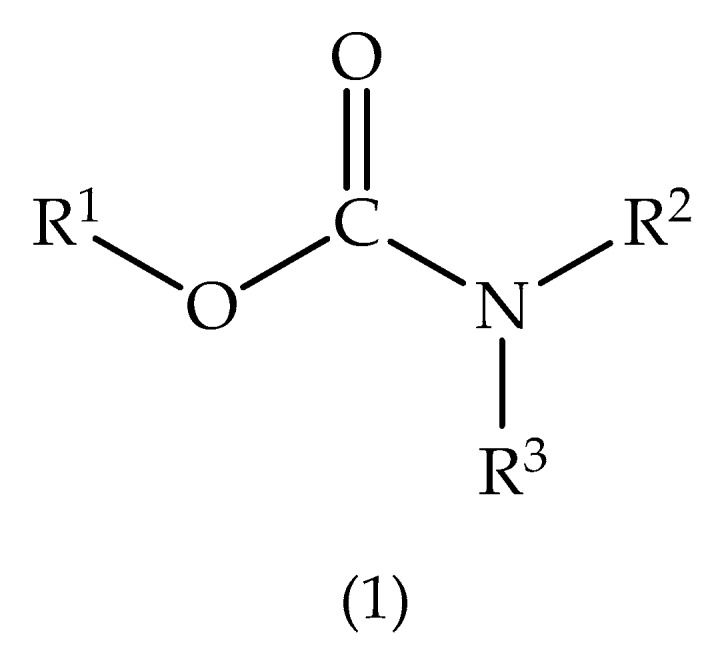
General structure of carbamates.

Along with OP, the carbamates (Figure 4) stand for the major class of insecticides involved in poisoning. These compounds inactivate AChE and BChE enzymes, resulting in elevated ACh levels and leading to an acute cholinergic syndrome, with the emergence of muscarinic, nicotinic, and CNS signs and symptoms. These manifestations are dependent on the dose and route of exposure involved. OP agents easily cross the blood–brain barrier (BBB), while carbamates do not effectively penetrate the CNS. The employment of carbamates results in less neurological toxicity, and these substances do not accumulate in the body [41].

**Figure 5:**
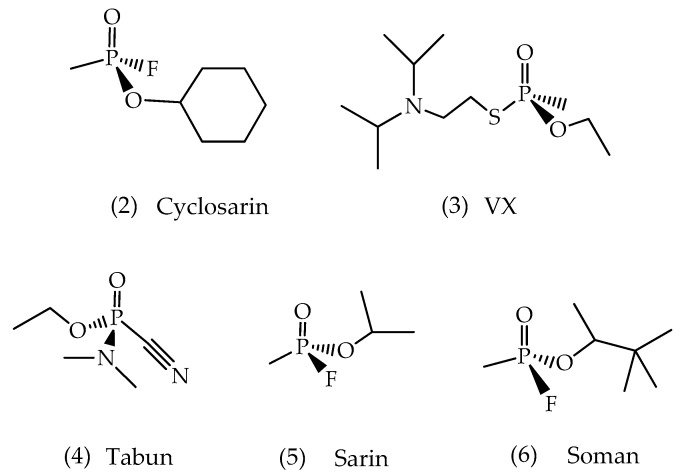
Chemical structures of the main warfare nerve agents.

Nerve or neurotoxic agents are the most lethal group among the phosphorus-based compounds [42]. The most well-known are shown in Figure 5. They have a chiral phosphorus atom that generates a pair of optical isomers in equal proportions. Soman, also having an optically active carbon in the pinacolyl group, gives rise to another pair of enantiomers. Although this agent has two stereocenters [43], it is known in the literature that the dominant chiral center is the phosphorus atom as it determines the potential toxicity of this compound. Therefore, this agent is broadly employed for theoretical and experimental investigations [44].

**Figure 6:**
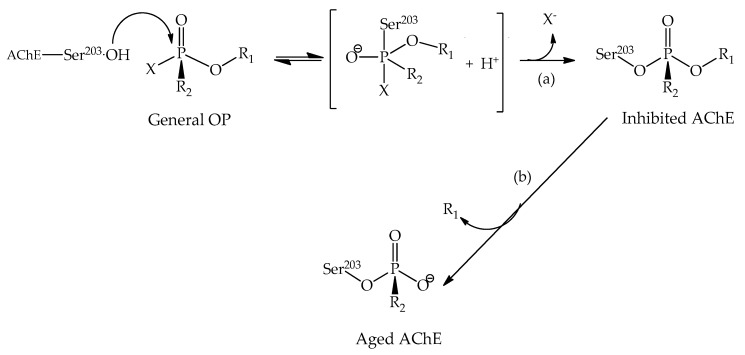
General representation of the (**a**) inhibition and (**b**) aging mechanisms [47].

Besides insecticidal agents, chemical warfare agents stand for an even more toxic form of OP. The first large-scale application of these OP chemical weapons occurred during World War II, and from then on, a range of compounds has been developed, presenting high toxicity and hazardousness [41]. These extremely toxic molecules are considered a serious threat to national security due to their potential use in terrorist actions. Although these chemical warfare agents were banned by the International Chemical Weapons Convention (CWC), numerous synthetic pathways for various nerve agents have been reported, and huge stocks are still available [41]. Due to their chemical reactivity, these OP agents, over the long term of human exposure, have the potential to toxically compromise the nervous system, causing several life-threatening serious diseases [45,46]. The inhibition and aging mechanisms are shown in Figure 6.

According to Figure 6, this reaction is governed by the phosphorylation of the hydroxyl functional group [48,49]. The AChE inhibition process takes place within the active site through a biomolecular nucleophilic substitution (S_N_2) reaction, with the formation of a covalent bond between the oxygen atom of serine and the central phosphorus atom of the OP [50]. After a period that varies according to the neurotoxic agent, the enzyme may be inhibited in an irreversible process known as aging (Figure 6), which is characterized by a dealkylation process [47]. Spontaneous reactivation of the enzyme may also occur, but at a practically insignificant rate [51]. 

**Figure 7:**
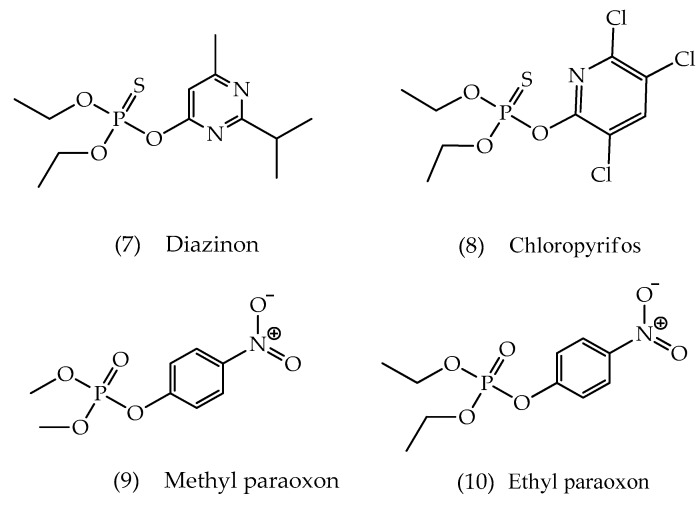
Chemical structures of some important pesticides.

Neurotoxic OP agents are substances that have several chemical and biological properties, such as high toxicity, lipophilicity, volatility. These properties favor the rapid insertion of these compounds through the airway and topical by allowing for the absorption of a reasonable concentration, as well as making them able to penetrate the BBB. In addition, the easy manufacture and low cost favor the use of some compounds of this class as agrochemicals [52]. Among the agrochemicals marketed, the OP agents were found to be the most preferred ones due to their wide spectrum bioactivity and easy availability. Some examples are ethyl paraoxon, methyl paraoxon, diazinon, and chloropyrifos (Figure 7) [41].

**Figure 8:**
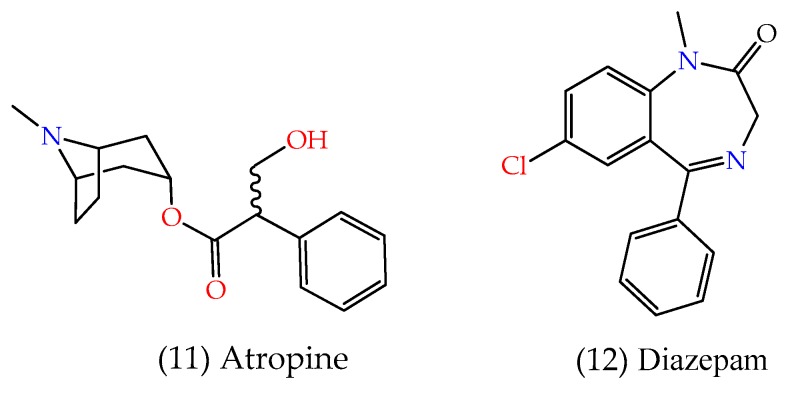
Representation of the chemical structures of atropine and diazepam.

In general, the resource adopted against neurotoxic poisoning proposes the use of atropine, diazepam (Figure 8), and an oxime [53,54]. This process will be better discussed in the next topic.

Given the great utility of AChEI in medicine and the limited therapeutic arsenal for the treatment of neurodegenerative diseases, such as Alzheimer’s disease (AD), as well as the problems related to this therapy [55], the evaluation of the potential of suitable compounds in inhibiting or reactivating AChE has great relevance for the development of new drugs [26]. 

## 4. AChE Reactivation Processes

**Figure 9:**
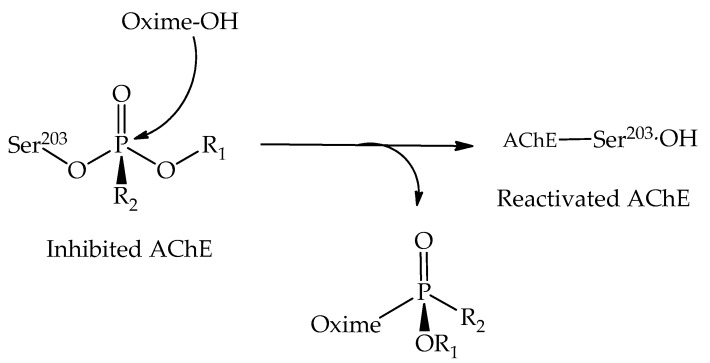
General representation of the reactivation process of the inhibited acetylcholinesterase (AChE).

Reactivators are nucleophilic substances able to cleave the covalent bond formed in the inhibition process between OP and AChE, thus restoring the enzyme catalytic activity. Some challenges still remain for future studies. Pralidoxime, discovered in 1955, was the first effective reactivator to be used as a drug in the treatment of OP intoxication, being one of the most widely used reactivators to date [56,57,58]. Note that each OP residue formed after inhibition differently modifies the enzyme active site, creating a selectivity of reactive molecules with the potential to bind to the enzymatic cavity [59,60]. The reactivation mechanism takes place basically in two stages: The first one is the approximation to the phosphorus atom of the AChE-OP adduct by the reactivator, forming a pentacoordinate transition state. The second step is the release of the OP-reactivator conjugate, restoring the enzyme function (Figure 9) [61,62]. 

**Figure 10:**
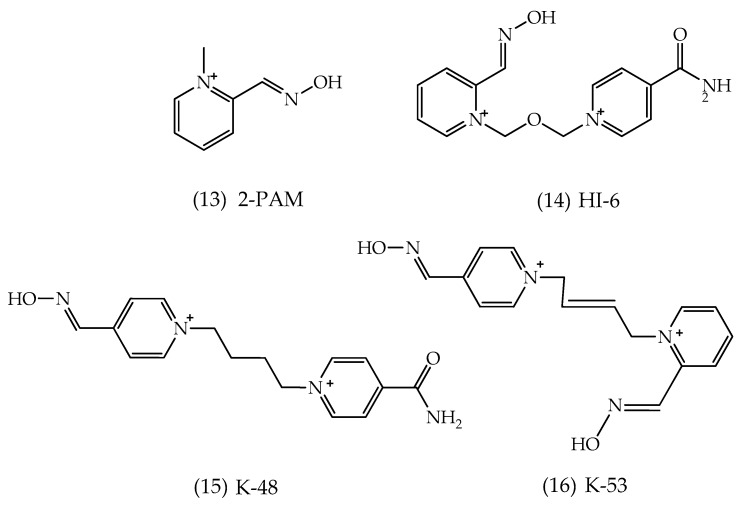
Representation of the chemical structures of oximes.

Reactivators generally may have one, two, three, or no pyridine ring. In general, quaternary pyridine rings interact with AChE peripheral anionic site (PAS), by stabilizing the reactivator in the cavity and favoring the nucleophilic attack. Some factors listed in the literature that may influence the reactivator efficiency are the presence of quaternary nitrogen in the reactivator structure; in case of bis-pyridinium oxime, the length and rigidity of the connection chain between them; the presence of the oxime functional group; the position of the oxime group on the pyridinium ring; and the number of nucleophilic groups in the reactivator structure [63]. The first reactivators to be studied were from the oxime class. Oximes are compounds that have at one end the functional group -C=N-OH, and in biological environments, they are usually found in the conjugate base form, the oximate ion. Most of the time, oxime molecules may contain one or two oxime functional groups, such as 2-PAM (also known as pralidoxime) and HI-6 (also known as asoxime), both widely marketed (Figure 10) [56,64]. 

Over the past 50 years, the class of oximes has been exhaustively studied. Although some molecules have good efficiency with some specific OP, there are no broad-spectrum antidotes yet. It is important to mention that, generally, quaternary oximes have a permanent positive charge, which interacts with PAS, thus increasing the related efficiency. However, this charge makes it difficult for the passage of the molecule through the BBB. This implies a divergent observation between good in vitro test results and not satisfactory in vivo test results [60]. 

Several recent works aimed at improving current antidotes by studying factors that may boost their efficiency and increase their crossing through BBB. Kuca and co-workers, for example, in in vitro tests with aryloximes and some neutral derivatives, have shown promising data on the effect of substituents, either electron-donating or electron-withdrawing with potential for existing oximes improvements, such the reactivator’s potentiality as pharmacokinetics [65]. In the same line of reasoning, in a study with bisoxime, a three positive charge oxime, it was shown that even with its hydrophilicity, the reactivator was able to be stabilized in the Sarin and VX-inhibited AChE cavity through interactions with some amino acid residues, such as Tyr124 and Tyr337. Despite this finding, due to the three positive charges, this reactivator would not penetrate the BBB. However, it is an option for BChE reactivation. This enzyme is a bioscavenger for the detoxification of free nerve agents in the body [66]. 

Another study showed the influence of the non-nucleophilic end position on isomeric oximes, showing a large difference in the reactivation rate of each isomer in relation to AChE inhibited by different nerve agents [67]. The change in position of the carbamoyl group resulted in different reactivation percentages for different enzyme–nerve agent complexes. With the aid of theoretical studies, it was evidenced that the structural changes between the isomeric oximes resulted in different interactions with the cavity residues. In addition, one of the isomers’ transition states was able to be better stabilized, especially in the cavity of the AChE-tabun cavity [67,68]. Diverse oximes-related aspects, such as interaction within AChE and reactivity, have been theoretically investigated [69]. Another approach by the same authors is the use of oximes as a pretreatment prior to acute paraoxon poisoning. The work concluded that the tested oximes, such as K-48 and K-53 (Figure 10), are able to significantly reduce the risk of death of the tested individuals when applied pre-emptively before pesticide poisoning. The tested hypothesis of using these molecules as pretreatment may be an alternative to complement the current treatment protocol [70].

Even with the benefit of oximes for the treatment of OP poisoning, as exposed previously, there is a concern about the balance between applied concentrations and the reactivator´s efficiency without causing tissue damage to the treated organisms. In their work, Kuca and coworkers studied such effects of varying concentrations of reactivators and the damage done to certain living tissues. As found in the literature, it was confirmed that all reactivators tested at high dosages caused damage to both visceral tissues and the overall health of the individual. The data obtained may be an important support for dose choices regarding different reactivators structures for newly developed drugs [71,72]. 

**Figure 11:**
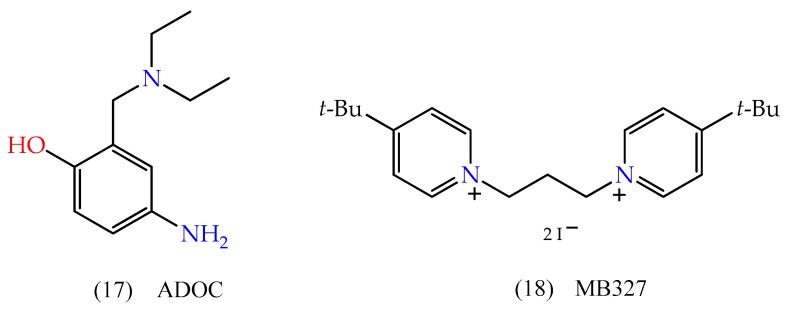
Chemical structures of 4-amino-2-(diethylaminomethyl)phenol (ADOC) and 1,1’-(propane-1,3-diyl)bis(4-tert-butylpyridinium)di(iodide) (MB327).

Although oximes are the main class of reactivators, several research groups are investing efforts in other non-oxime molecules. Katz et al. (2015) [73] screened a series of compounds with the potential to reactivate OP-inhibited AChE. In this regard, it highlights 4-amino-2-(diethylaminomethyl)phenol (ADOC), which showed some interesting results. This molecule piqued the interest of other groups that studied structural variations and substituent groups of its structure, such as from Konig et al. (2018) [74] and Cadieux et al. (2016) [75]. Another interesting group of molecules under study comes from tert-butyrylpyridinium, with emphasis on 1,1’-(propane-1,3-diyl)bis(4-tert-butylpyridinium)di(iodide) (MB327). Niessen et al. (2018) [76] studied the efficiency of MB327 and some of its regioisomers, finding some important results in soman-inhibited AChE reactivation. The chemical structures of ADOC and MB327 are displayed in Figure 11. Zhuang et al. (2018) [77] also screened a series of compounds derived from quinone methide precursors, that in vitro tests were able to reactivate OP-inhibited AChE, in addition to resurrecting aged-AChE. Regarding the AChE reactivation, the review from Gorecki et al. (2016) [78], which covers remarkable developments since the discovery of 2-PAM, in 1955, should be mentioned. 

## 5. Update of the Recent Patents in Literature from 2016 to 2019

Faced with all exposed so far, there is an attempt to develop effective ways of reactivating AChE through specific reactivators [60]. In this context, this becomes a wide field of study, with some recent patents already produced. Some of them are described ahead in this review.

Currently, the protocol for an effective treatment of the intoxication by neurotoxic OP consists of the previous knowledge about the kind of OP from which the exposure has occurred. This situation is not easy to address in a mass-exposure event because of the short time for the patients to develop the intoxication symptoms. Therefore, quick treatment is strongly required [60]. This fact justifies the efforts made to develop novel efficient oximes and reactivators capable of treating multiple OP neurotoxic agents. However, the high complexity levels of the enzyme active site, after inhibition by diverse OP inhibitors, make necessary different therapeutic approaches owing to the size and orientation of the attached O- or N-alkyl groups [59,79,80]. 

**Figure 12:**
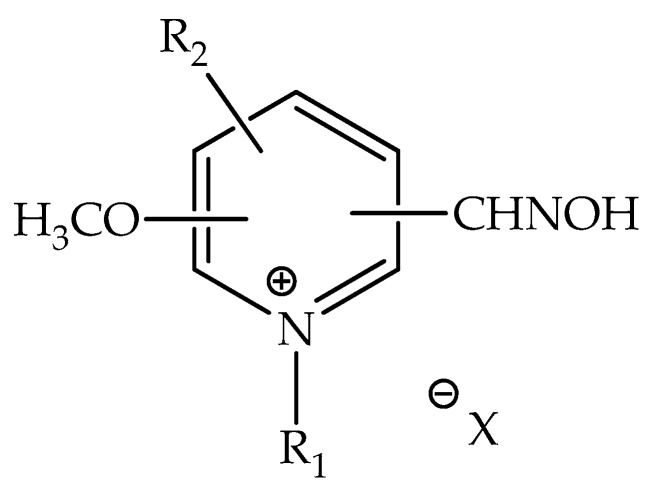
Formula I of the compounds developed in this patent.

The patent from Quinn and Topczewski, published in 2016 under the Pub. No.: US 2016/0151342 A1 [47], provides compounds and methods that can be used for applications in reactivation processes of the aged-AChE adduct. It is useful to counteract the intoxication caused by OP nerve agents. Regarding the employment of oximes, if administered shortly after exposure, the oxime is capable of displacing the bound OP, thus releasing the serine residue. However, as discussed previously, if the administration is delayed, a process called aging takes place by the occurrence of a solvolytic loss of an alkyl group from the AChE-OP adduct. The aged adduct is stabilized by diverse interactions with the enzyme active site, turning out to be ineffective to oxime reactivation [81]. To date, there is a lack of antidotes against aged AChE-OP adducts, but scientists worldwide are striving to discover new potent antidotes for this end [82]. Based on the exposed so far, this patent brings about the discovery of a class of molecules that can reactivate an aged AChE-OP adduct, whose process is denominated by resurrection. The method from Quinn and Topczewski can lead to the adduct reactivation with the use of alkylating agents. The invention consists of a method for treating a mammal suffering from OP intoxication, by administrating an alkylating agent, which can be performed along with a possible joint administration of ACh receptor antagonist and/or anti-seizure agent. The respective compounds discovered possess the general formula shown in Figure 12. 

**Figure 13:**
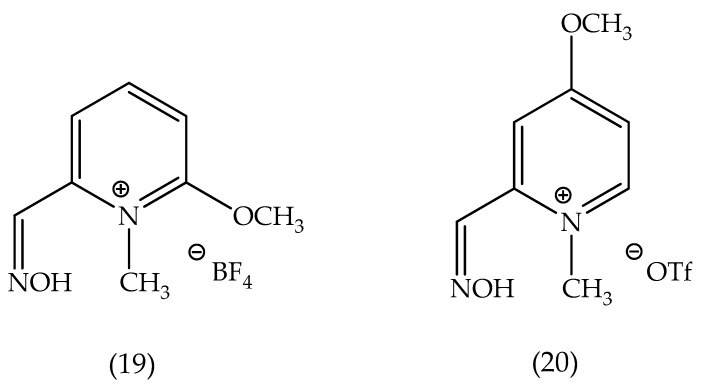
Representation of the chemical structure of some molecules synthesized.

According to that formula, a range of molecules is possible to be synthesized. Realize that X (Figure 12) can be any suitable counter ion. R_1_ and R_2_ stand for a range of chemical groups, thus allowing for the development of different oxime molecules. The formula I-based molecules can be formulated as pharmaceutical compositions, with a posterior administration to a mammalian host, such as human beings. The administration can be adapted according to the chosen route, such as orally or parenterally, by intramuscular, intravenous, topical, or subcutaneous routes. Appropriate dosages of the compounds from Formula I can be determined by making a comparison regarding their in vitro and in vivo activity in animal models. The patent describes the synthesis route for some molecules, such as the molecules highlighted in Figure 13. As a matter of fact, these molecules have suggested good potential for applications in the resurrection process of the aged-AChE. The synthesis of N-methyl-methoxypyridinium can occur through the exposure of starting pyridines to trialkoxonium tetrafluoroborate (or another alkylating agent, such as methyl triflate (MeOTf)) in an appropriate solvent before or after the oxime formation. For instance, this patent brings about promising experimental results by employing Compound **19**, demonstrating its good performance for reactivation. 

In this experiment, the human AChE (*Hss*AChE) was inhibited by exposure to an OP agent analog of Sarin, 7-(isopropyl methylphosphonyl)-4-methylumbelliferone, being incubated with Compound **19** and 2-PAM. The experimental results are displayed in Table 1. Note that the best results were achieved in larger periods of incubation. 

The aging process was considered irreversible in the past decades, but many attempts have been made to convert the phosphorylated oxyanion aged form to a re-alkylated form of AChE. According to Franjesevic et al. (2019) [60], the reactivation or re-alkylation processes of aged AChE are quite challenging. At first, a substrate must bind selectively and efficiently in the enzyme active site, in an active conformation, so that it can facilitate the critical transition state for the desired re-alkylation, thus allowing for the re-alkylated phosphorylated serine to be reactivated by an efficient nucleophile. It is important to notice that all of these steps should occur inside an active site that is relatively compact and constricted. The aged state of AChE is a thermodynamically stable form of the enzyme, and the resurrection process may show some difficulties in overcoming the conformational changes and hydrogen-bonding networks within the active site of aged adduct [60].

**Figure 14:**
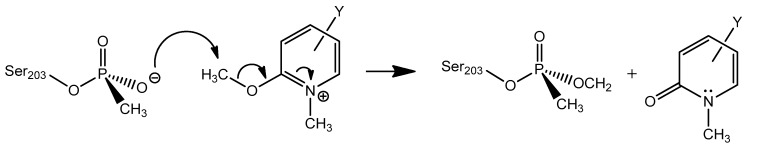
Proposed resurrection process for aged AChE.

Quinn et al. (2017) [83] demonstrated that the aged state of the enzyme makes four hydrogen bonds. One of these interactions is with the aged serine residue, between the phosphorylated oxyanion and the histidine residue, and the others with the oxyanion hole. A good stabilization of the tetrahedral intermediate in the deacylation step is observed. The action of a successful resurrector of aged AChE is crucial. This active species has to bind to disrupt the hydrogen bonds formed in the aged state and cause relevant conformational changes within the active site. These steps are important for reducing the strength of the intermolecular interactions, to allow for reactivity of the phosphorylated oxyanion. This makes possible the desired transition state for electrophilic re-alkylation. Figure 14 stands for a reaction scheme of the resurrection process of the aged AChE, proposed by Quinn and co-workers [83]. 

**Figure 15:**
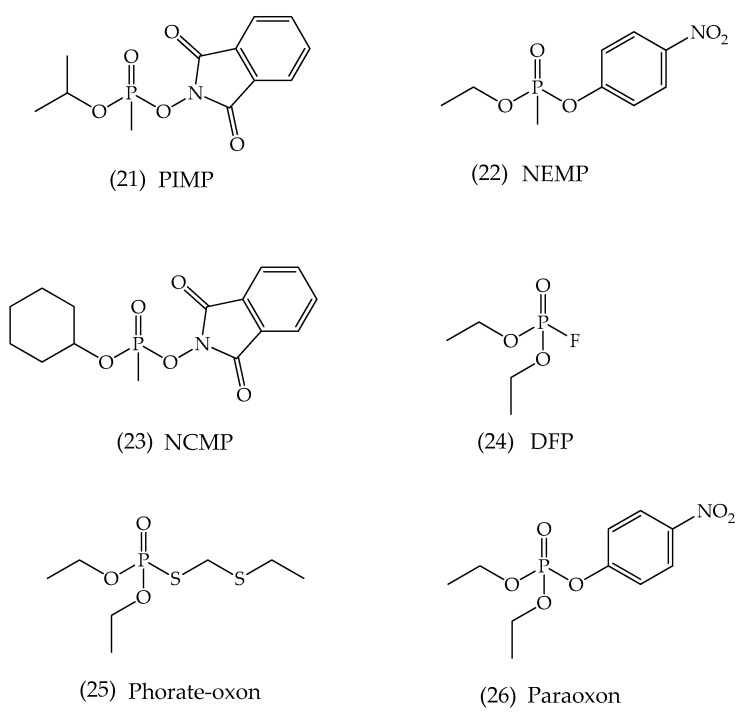
Representation of the chemical structures of some organophosphorus (OP) used in the patent.

Regarding the reactivation processes of OP-inhibited cholinesterase enzymes, the BChE enzyme also stands out. The patent from Chambers at al. (2017) [84], published under the Pub. No.: US 2017/0258774 A1, brings about novel oximes for reactivating the OP-inhibited BChE. The authors state that the oxime molecules can allow for a dual reactivation in the treatment process, which can take place by reactivating both serum BChE and inactivated CNS AChE. This invention lies in the field of nerve agent antidotes’ development and protection against the toxic effects caused by these toxic OP inhibitors, nerve agents, and/or pesticides. For these antidotes, the administration can be performed intravenously, intraperitoneal injection, orally, nasally, topically, among many others. The researchers synthesized a range of phenoxyalkyl pyridinium oximes, according to previous work from Chambers et al. (2016) [85]. By following the line of investigation adopted, many of these oximes were employed to determine their ability to reactivate inhibited serum BChE by nerve agent surrogates and insecticidal oxons. In this line, the inhibitors investigated were PIMP (phthalimidyl isopropyl methylphosphonate, sarin surrogate), NEMP (nitrophenyl ethyl methylphosphonate, VX surrogate), NCMP (nitrophenyl cyclohexyl methyl phosphonate, cyclosarin surrogate), and DFP (diisopropyl fluorophosphate), along with two insecticidal OP: paraoxon and phorate oxon, which are metabolites of the insecticides parathion and phorate, respectively (Figure 15). 

The authors demonstrated that the oximes evaluated were sufficiently capable of crossing the BBB, thus being effective as soon as these species enter the brain. The PNS is part of the nervous system that consists of nerves and ganglia on the external environment, outside of the brain and spinal cord, thus not being protected by the BBB. On the other hand, the CNS consists of the brain and spinal cord, being protected by this barrier. The present oximes showed to be more effective antidotes for OP intoxication. One feature of these oximes is the fact that they present increased lipophilicity, with the aim of enhancing the ability of these molecules to cross into the brain. By targeting BChE, crossing through the BBB does not become a great issue. On the other hand, the fact of these oximes present dual reactivating properties makes them capable of reactivating AChE in CNS and BChE in the circulatory system. With all exposed so far, the most interesting is that these molecules can scavenge and destroy circulating OP that potentially could inhibit AChE and lead to intoxication. 

Based on a range of substituted phenoxyalkyl pyridinium oximes, their goal was to identify some of these molecules that also have the potential to reactivate BChE. However, very interestingly, this enzyme can provide some protection by being inhibited by the OP neurotoxic agents. Note that this inhibition is stoichiometric, and one OP molecule is destroyed for every BChE inhibited. However, the related protection is limited by the amount of BChE present in the serum. In this patent, the authors investigated whether the BChE can be reactivated and the active site restored so that the BChE could be inhibited again, thus destroying another OP molecule. In this context, the ability of BChE to protect from intoxication could be enhanced. It is worth mentioning that the best oxime molecules from this work exhibit activity toward both AChE and BChE, thus presenting protective effects by displaying two therapeutic mechanisms, providing a substantial improvement for currently-approved antidotes. 

**Figure 16:**
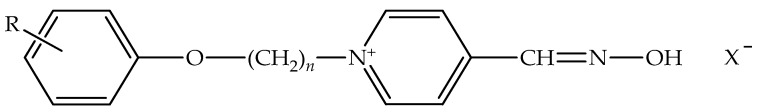
Representation of the general chemical formula for the oxime molecules investigated.

The oximes developed, according to Chambers et al. (2016) [85], display the ability to counteract the harmful effects of OP poisoning through the restoration of the inhibited AChE in the peripheral and central nervous systems. Some of these oximes are shown to reactivate BChE as well. The identified reactivating molecules then restore the BChE activity, and multiple OP molecules could be destroyed. For that study, the serum BChE from human, guinea pig, and rat was used. The oxime molecules investigated share the common formula shown in Figure 16. 

Wherein R = hydrogen, alkyl, alkenyl, aryl, acyl, nitro, or halo; n is an integer selected from 3, 4, or 5; and X is a pharmaceutically acceptable anion. 

The phenoxyalkyl pyridinium molecules and methods of this investigation provided the military with a more efficient antidote against the intoxication caused by neurotoxic OP agents. Civilians can directly benefit from protection against terrorist attacks and OP pesticides-based poisoning. The authors showed significant broad-spectrum capability with the novel oximes to reactivate both AChE and BChE after exposure to these nerve agents. See Table 2 for more details about the chemical structures of the molecules investigated. The tested oxime molecules differ in the alkyl linker chain length (n) and/or the phenoxy ring substitution moiety (R).

According to the results of the investigations from Chambers et al. (2017) [84], diverse experimental essays were performed through BChE with different OP agents and oximes. The experimental essays with paraoxon-inhibited BChE show that the novel oximes exhibited a reactivation range of 33–72%, while 2-PAM averaged 32%. In this case, all oximes presented better results in relation to 2-PAM, except OX99, whose reactivation percentage was similar to that of 2-PAM. The only difference regarding OX98 is the alkyl linker chain length (from 4 to 5), and this small modification seems to be enough to significantly decrease the efficiency of OX98. On the other hand, this difference in efficiency between OX98 and OX99 was smaller for the reactivation of the PIMP-inhibited BChE. This suggests that the enzyme active site can give rise to different conformations dependent on the type of bound OP agent, thus interfering with the interaction modes of these oximes. It is important to notice that for better performance on these oximes in the reactivation process, they should adopt an appropriate and/or correct conformation in the site. For PIMP-inhibited AChE, the new oximes presented a range of 45–73%, and an average of 46% from 2-PAM. In the case of employing NEMP as the inhibitor, the reactivation range was 18–62% (novel oximes), and an average of 8% (2-PAM). According to this experimental essay with NEMP as an inhibitor, OX98 and OX99 showed similar efficiencies, however both were inferior in relation to those of the other oximes. The oximes (OX14, OX12, OX28, OX31, and OX59) showed the best results, which were remarkably superior to that of 2-PAM. For phorate–oxon-inhibited BChE, the new oximes did not bring about good results of reactivation, with the reactivation percentage from 2-PAM being noticeably superior. In this line, the accomplishment of further studies, such as theoretical ones, to better comprehend the interaction modes and reactivity of these oximes through BChE inhibited by different OP agents is necessary. According to the reactivation experimental essays of the BChE inhibited by NCMP and DFP, all new oximes and 2-PAM did not show a good performance for reactivation. These examples show a better performance of many of the developed oximes against OP inhibitors, in comparison with the traditional 2-PAM. However, the performance of each oxime may deeply shift dependent on the type of bound OP agent. 

Kovarik et al. (2006) [86] observed that the reactivation of phosphorylated BChE is not as successful with two well-known oximes, 2-PAM and HI-6, in comparison with the phosphorylated AChE. In addition, Musilova and co-workers investigated more than 20 oximes against AChE and BChE enzymes, both inhibited by the pesticide paraoxon [87]. The researchers demonstrated that none of the oximes were capable of reactivating BChE more efficiently than AChE, and this trend was also reported for diverse other OP agents bound to AChE and BChE [88,89]. This fact shows the importance of this patent, which provides novel therapeutic agents that bring about good results in reactivating both enzymes. 

**Figure 17:**
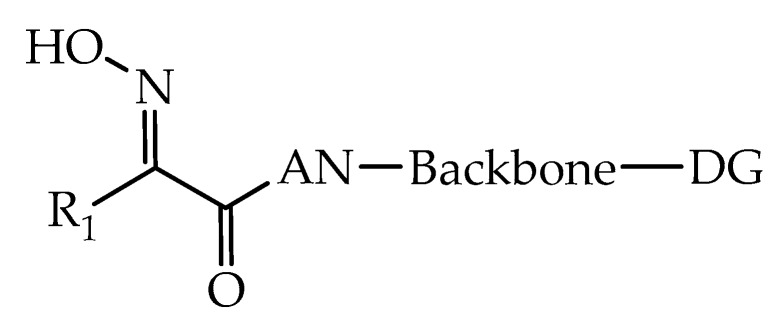
Representation of the general formula of oxime [90].

The current standard of care for exposure to OP-based AChEIs has changed very little over the past half-century. Accordingly, effective reactivation of OP-inhibited AChE and inactivation of OP-based AChEIs are still highly desirable and challenging. In this context, Valdez et al. (2019) [90] described a series of oxime-derived compounds capable of inactivating OP-based AChEIs (Pub. No.: US 2019/152920 A1). Figure 17 shows the general formula of oxime described by Valdez and coworkers. 

**Figure 18:**
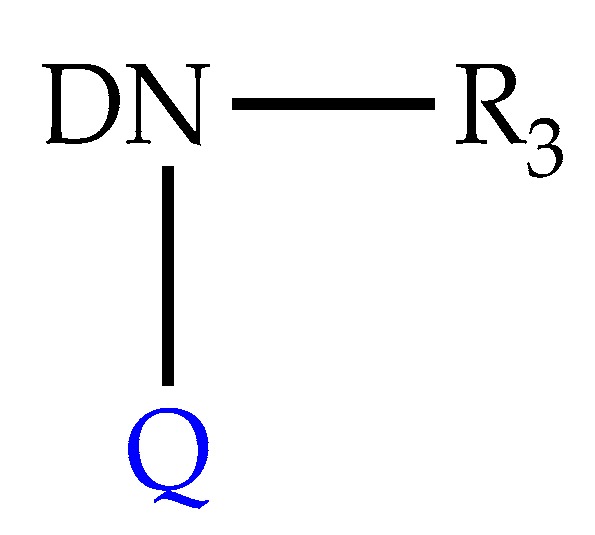
Representation of Formula II developed in the patent [90].

Wherein R_1_ = H or CH_3_; AN = amide nitrogen; Backbone = chemical moiety of at least two carbon atoms linking together AN and DG; DG = distal group containing a bicyclic moiety represented by Formula (II) (Figure 18). 

Wherein DN is nitrogen on a bicyclic core; R_3_ is a hydrogen, heteroatom, functional group, or a substituted or unsubstituted linear or branched alkyl chain, aromatic or aliphatic cyclic group; Q is a heteroatom or carbon atom on the bicyclic core.

Throughout the studies from Valdez and coworkers [90], a number of related methods, systems, and compositions for inactivating one or more OP-based AChEIs, as well as the therapeutic and/or prophylactic treatment of an individual and/or decomposition of OP-based AChEIs for decontamination [90], is listed. 

**Figure 19:**
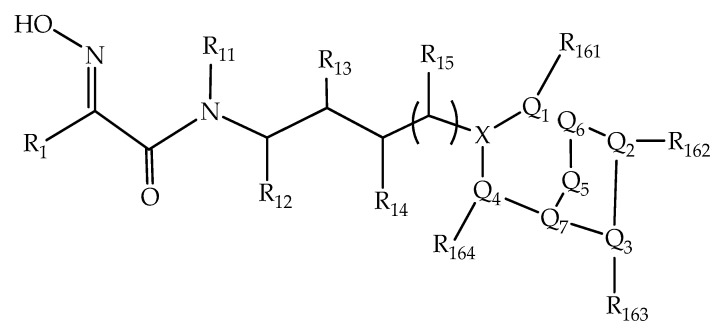
Formula III of an oxime developed in the patent [90].

In detail, the authors described Formula III (Figure 19), which is one of the oximes developed in the patent. Through this formula, diverse tests have been performed to reactivate the AChE enzyme and inhibit the OP agents. The authors also described a composition consisting of an effective amount of oxime described in the patent and a saline solution to reactivate the OP-inhibited AChE, along with the description of a method for treating, preventing, and decontaminating an individual’s exposure to neurotoxic OP [90].

Wherein X = N or C; R_10_, R_1_ = H, or CH_3_; R_10_, R_11_, R_12_, R_13_, R_14_, R_15_, R_161_, R_162_, R_163_, R_164_, and R_165_ = alkyl, alkenyl, alkynyl, aryl, arylalkyl, or alkylaryl groups; Q_1_, Q_2_, Q_3_, Q_4_, Q_5_, Q_6_, and Q_7_ = N, O, or S or saturated, unsaturated or an aromatic ring.

According to the patent, the synthesized compounds are able to penetrate the BBB, and this is a very important feature, keeping in mind that the oxime compounds thus have a more specific and efficient action. Investigations in this area arouse the interest of research groups for the potentiation of pharmacological action, physicochemical properties of these new compounds and to make them more effective in the reactivation process of AChE. However, in this patent, the oxime derivatives were the main reactivating agents used. Although these compounds are very ordinary in this area, it is necessary to search for a synthesis of new classes of compounds for this same purpose, the search for new strategies. 

The reactivators presented by Valdez and coworkers [90] in the present patent are demonstrated to reactivate both AChE and BChE enzymes. To reinforce its importance, note that BChE is involved in nervous system development, detoxification of natural and synthetic toxins, hydrolysis of drugs, such as cocaine, heroin, and aspirin, fat metabolism, as well as the interaction and functional modification of other proteins, such as polyprolines and trypsin [91,92]. 

**Figure 20:**
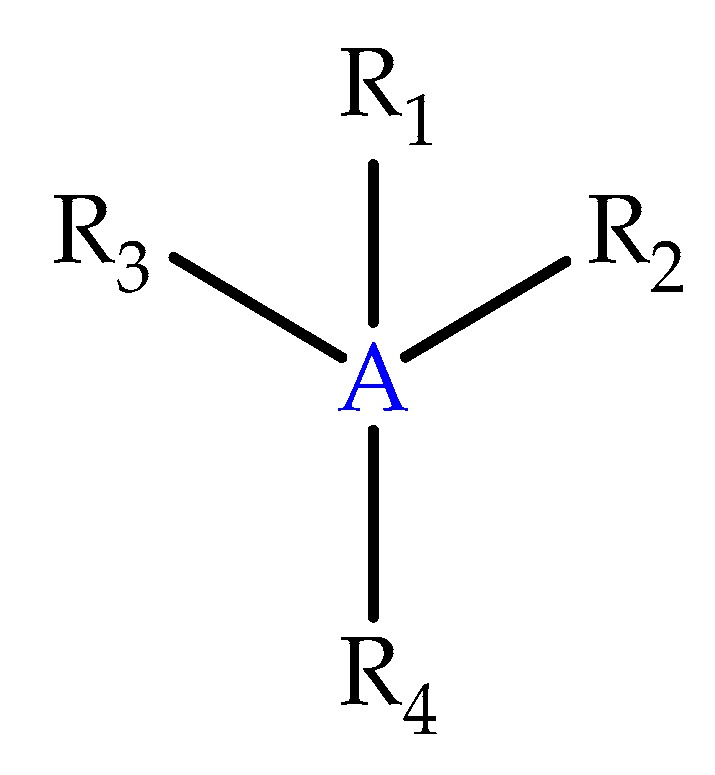
Formula IV for the novel molecules developed in the patent.

In 2017, Khavrutskii et al. (2017) [93] invented compounds, compositions, and methods for activating, reactivating, reversing, or preventing the deactivation of AChE and BChE, with compounds derived from Formula IV (Pub. No.: WO 2017/218886 A1, Figure 20): 

Wherein A is a 5- or 6-membered substituted or unsubstituted aromatic ring, cycloalkyl or heterocyclic ring; R_2_ and R_3_ is H,CN,OR_10_, S(O)_0-2_R_10_, NR_11_R_12_, C(O)NR_13_R_14_, C_1-6_ alkyl, and C_1-6_ alkene; R_4_ is H, CN, OR_10_, S(O)_0-2_R_10_, C_1-6_ alkyl, and C_1-6_ alkene, NR_11_R_12_ and C(O)NR_13_R_14_; R_5_ is C_1-4_ alkyl, and C_1-4_ alkene; R_6_ is H and C_1-3_ alkyl; R_7_ is substituted or unsubstituted C_1-3_ alkyl and substituted or unsubstituted C(O)OR_15_; R_8_ is C(O)NHR_15_ and C(O)OR_15;_ R_9_ is H, CN, C(R_6_)OH, OR_8_, NR_9_R_10_, C(O)NR_11_R_12_, C_1-6_ alkyl, and C_1-6_ alkene; R_10_ is H, C_1-3_ alkyl, and C_6-12_ aryl; R_11,_ R_12,_ R_13,_ and R_14_ is H, C_1-4_ alkyl, and C_1-4_ alkene; R_15_ is H and C_1-3_ alkyl.

The methods disclosed by the authors of this invention relate to the administration of at least one compound of the composition comprising Formula IV. Specifically, the methods include reactivation, deactivation, or prevention of AChE or BChE processes. The methods of the present invention include treating a subject for toxicity associated with AChE inactivation, with the methods comprising administering a therapeutically effective amount of at least one compound of Formula IV [93].

It is important to highlight that this patent focused on the synthesis of new antidotes, modifying the mechanism of action, reactivation process agents for both AChE and BChE. This is clearly a new and promising strategy; however, the patent does not mention whether the synthesized compounds penetrate the BBB and whether these new antidotes are more efficient than oxime compounds.

In general, when comparing the classes of reactivators used in the patents described here, all molecules have nucleophilic groups or a quaternary nitrogen. It is known that these pharmacophoric groups interact with the “anionic” catalytic site, by leaving the nucleophile in the opposite position to the phosphorus atom to attack and displace the phosphoryl group of the inhibited enzyme. Particularly, in the patent by Valdez et al. (2019), the binding of the quaternary N occurs within a ring. This fact is very interesting since cycles are limited to the rotation of the molecule, thus reducing the number of conformations that the molecule can adopt. Thus, the molecule is more likely to be in an active conformation as it approaches the enzyme active site. Compounds containing these pharmacophoric groups are potent reactivators of AChE [63,94]. 

## 6. Expert Commentary

The intoxication caused by neurotoxic OP agents has increasingly become a serious public health concern around the world. Many researchers have strived to find novel therapies for reversing the poisoning caused by these agents. In this context, the chemical agents denominated reactivators are the most used in the treatment protocol, along with the administration of a muscarinic receptor antagonist, such as atropine, to stop nerve receptor overstimulation. In addition, diazepam is also used as an anticonvulsant to prevent brain damage and minimize the central side effects due to nerve agent-induced incapacitation. To date, there are no broad-spectrum reactivators able to reverse the intoxication caused by different OP agents. In the current treatment protocol, previous knowledge about the kind of neurotoxic agent that the patient has been exposed to is required. Due to the intoxication level, and the speed by which the symptoms of the poisoning progress, immediate treatment is necessary. Most times, it is not possible to know the kind of OP agent the victim has been exposed to, making the administration of an antidote kit necessary. In an ideal situation, there would be a universal antidote, which would be active against a range of OP agents, and many researchers are making efforts in the search for it. 

The patents analyzed employed in vitro and/or in vivo studies to investigate the performance of these molecules in reactivating cholinesterase enzymes. A more complete and reliable analysis consists of both experimental essays, given that the likely good results obtained in vitro may not be reproduced in vivo. This comes from the fact that in in vivo studies, the new compounds may find difficulties to cross the BBB, thus turning out to be inefficient. Regarding the patents, the authors also brought about a discussion concerning the administration forms of the novel antidotes, as well as their effective dosages. This analysis is important because the administration of an inappropriate dosage of these agents can result in toxicity and damages to health. In this review, we could observe different patents, which approach interesting mechanisms of action. In this context, the new antidotes may perform the reactivation of both AChE and BChE, along with the alkylating agents that allow for the reactivation of the aged adduct. These research lines are an important starting point to the discovery of promising or even universal antidotes, for a rapid and complete detoxification process. 

The discovery of more effective therapies is essential to provide better and more efficient treatments for intoxicated patients. Further investigations are required to better understand the performance and mechanism of action of the novel reactivators under development. The patents analyzed show a big advance in this area, but the limited amount of registered patents shows the difficulty of getting promising and efficient reactivators. These patents present very promising reactivating compounds, and their therapeutic approaches can undoubtedly provide novel means to combat the poisoning caused by OP neurotoxic compounds. Due to recent advances in this field, an increasing number of works, even new patents, over the next years is expected. The development of novel antidotes for OP intoxication is crucial due to the frightening numbers of intoxicated people annually in the world, majorly due to the misuse of agrochemicals. In the case of poisoning, quick detection and identification of the toxic OP agent are crucial for effective protection, considering the lack of broad-spectrum reactivators. 

With regard to potential drug candidates, the reactivators investigated in these patents still suffer from the lack of biological activity data, as well as physical–chemical and mechanistic parameters. More studies become necessary to push these compounds forward, making them even more effective as reactivators. The lack of more deep in vivo studies maintains these drug candidates at the experimental level only [95]. 

## 7. Conclusions

The role of AChE for the proper functioning of the nervous system and processes involved is leading to an increase in researches about this enzyme. The current scenario marked by the use of OP agents in terrorist attacks, coupled with the increasing number of poisonings caused by the misuse of these compounds, has alerted the scientific community about the importance of studies directed to this area. For this purpose, this review has detailed the current patents related to novel cholinesterase reactivators. 

The several damages caused by these toxic substances and the ease of use justify the search for the development of more efficient defense compounds against neurotoxic agents, especially OP compounds. The complexity of the inhibited AChE enzyme active site makes different approaches necessary due to the size and orientation of their specific groups. Still, as observed in this review, very important advances have been made in this field. Thus, the expectation would be to establish a treatment capable of providing coverage against a variety of OP agents. 

The patents evaluated address primordial points that, in fact, contribute to the evolutionary process of the development of promising antidotes. Nowadays, the use of oximes is considered the most viable option, as they are capable of displacing the OP, thus releasing the serine residue and reactivating the enzyme. Among the described methodologies, we highlight the use of alkylating agents for aged-AChE reactivation, with joint administration of ACh receptor antagonist and/or anticonvulsant agent. The method from Quinn and Topczewski, which was detailed previously in one of the patents, points to the possibility of synthesizing a series of molecules that may contribute to the development of a range of oxime molecules. 

On the other hand, regarding the development of treatments targeting aged AChE, another patent about molecules that seem to have a favorable potential for the enzyme reactivation process, which was observed from significant experimental results, was detailed. At last, this review presents essential information that serves as a starting point for researchers in medicinal chemistry, as well as clarifying the existence of coherent works developed for the same purpose. Despite the lack of a universal antidote, the range of information available allows for the discovery of new and powerful remediation techniques. 

## Figures and Tables

**Table 1:** Resurrection essays data obtained in the experiment with inhibited *Hss*AChE (Pub. No.: US 2016/0151342 A1).

Time, Hours	$\%\;AChE_{React}$	$\%\;AChE_{aged.}$	$\%\;AChE_{Res.}$
1.00	2.45 (± 0.7)	1.16 (± 0.06)	1.28 (± 0.8)
4.00	5.67 (± 0.6)	1.16 (± 0.06)	4.50 (± 0.6)
12.00	6.46 (± 1.1)	1.16 (± 0.06)	5.30 (± 1.1)
24.00	8.70 (± 1.3)	0.00 (± 0.06)	8.70 (± 1.3)
48.00	7.03 (± 0.8)	0.00 (± 0.06)	7.03 (± 0.8)

**Table 2:** List of oxime molecules used in the patent (Pub. No.: US 2017/0258774 A1) [84].

BChE Reactivation Tested Oxime Molecules		
Tested Phenoxyalkyl Pyridinium Oxime Molecule	Alkyl Linker Chain Length (n)	Phenoxy Substitute Moiety (R)
Oxime 12 (OX12)	5	4-CH_3_ –O–
Oxime 14 (OX14)	4	4-Cl–
Oxime 28 (OX28)	4	4-CH_3_CH_2_C(:O)–
Oxime 31 (OX31)	3	3-CH=CHCH=CH-4
Oxime 32 (OX32)	4	3-CH=CHCH=CH-4
Oxime 59 (OX59)	4	4-Ph–CH_2_–O–
Oxime 98 (OX98)	4	4-(CH_3_)_3_CCH_2_C(CH_3_)_2_ –
Oxime 99 (OX99)	5	4-(CH_3_)_3_CCH_2_C(CH_3_)_2_ –
